# Transcrystalline Mechanism of Banded Spherulites Development in Melt-Crystallized Semicrystalline Polymers

**DOI:** 10.3390/polym16172411

**Published:** 2024-08-25

**Authors:** Theodor Stern

**Affiliations:** Department of Chemical Engineering, Biotechnology and Materials, Faculty of Engineering, Ariel University, Ariel 40700, Israel; theodorst@ariel.ac.il

**Keywords:** banded spherulites, transcrystallinity, nucleation, amorphous phase, chain orientation, lamellae, morphology

## Abstract

The decades-long paradigm of continuous and perpetual lamellar twisting constituting banded spherulites has been found to be inconsistent with several recent studies showing discontinuity regions between consecutive bands, for which, however, no explanation has been found. The present research demonstrates, in three different semicrystalline polymers (HDPE, PEG10000 and Pluronic F-127), that sequential transcrystallinity is the predominant mechanism of banded spherulite formation, heterogeneously nucleated on intermittent self-shear-oriented amorphous layers excluded during the crystals’ growth. It is hereby demonstrated that a transcrystalline layer can be nucleated on amorphous self-shear-oriented polymer chains in the melt, by a local melt flow in the bulk or in contact with any interface—even in contact with the interface with air, e.g., in contact with an entrapped air bubble or at the edges of the sample—or nucleated following the multiple directions and orientations induced by a turbulent flow. The bilateral excessive local exclusion of amorphous non-crystallizable material, following a short period of initial non-banded growth, is found to be the source of dislocations leading to spirally banded spherulites, through the transcrystalline layers’ nucleation thereon. The present research reveals and demonstrates the sequential transcrystalline morphology of banded spherulites and the mechanism of its formation, which may lead to new insights in the understanding and design of polymer processing for specific applications.

## 1. Introduction

Polymers have become some of the most predominant materials, as reflected in their vast variety of industrial products related to almost every aspect of modern life.

The vast majority of polymers, both synthetic and natural, are crystallizable and commonly exhibit a crystalline structure under ambient conditions [1,2,3,4,5,6,7,8,9]. As exceptions, some synthetic polymers lack the ability to crystallize, such as atactic polystyrene, poly(methyl methacrylate), polycarbonate and poly(vinyl chloride), which, consequently, are entirely amorphous and are glassy at room temperature [1,2,3,4,5,6,7,8,9].

In contrast to minerals, the crystallization of which commonly leads to the formation of faceted crystals, the crystallization of polymeric materials predominantly forms platelet-like crystallites, termed lamellae. Due to the characteristically very high molecular weights of the polymer chains, ranging from tens of thousands to hundreds of thousands and even to millions of g/mol, there are only two possible lamellar-forming crystallization mechanisms of polymeric chains. The first is the extended chain mechanism, in which the polymeric chains are parallelly oriented to each other. Polymers mostly do not naturally crystallize in an extended chain configuration, but it may be commonly induced by applying significant tensile stress or strong shear stress on the molten polymer, before and/or during the crystallization process. This mechanism is widely known in the industrial manufacturing of polymeric fibers using a spinning process [1,2,3,4,5,6,7,8,9]. The second is the chain folding mechanism—via which most polymeric materials naturally crystallize—which occurs by polymeric chain folding in the direction normal to the axial direction of the lamellae. The resulting lamellae can be of various lengths, widths, shapes and spatial configurations but are always of nano-scale thickness—commonly ranging between 10 and 20 nanometers in thickness—as governed by the polymeric chain fold length [1,2,3,4,5,6,7,8,9].

Melt-crystallized polymers predominantly consist of highly complex multi-lamellar crystals, which, when allowed to grow unobstructed, exhibit a quasi-spherical morphology and are thus termed spherulites [1,2,3,4,5,6,7,8,9,10,11].

Crystallizable polymers cannot completely crystallize and are thus typically semicrystalline, due to the inherent presence of non-crystallizable fractions, mainly originating from chain folding regions, chain entanglements, polydispersity and low chain mobility and diffusion. The non-crystallizable material is excluded during crystalline growth, accumulating around and coating all lamellar crystallites and filling all interlamellar gaps of the spherulites and outer surfaces thereof, and it constitutes the amorphous phase of the polymer [1,2,3,4,5,6,7,8,9]. Hence, semicrystalline polymers are considered nanocomposites, with the amorphous phase being the matrix (the continuous phase) and the lamellar crystals being the reinforcement (the discontinuous phase) [12,13]. Thus, the morphology, size and direction of the lamellar crystals within the amorphous phases of semicrystalline polymers may greatly affect their physical and mechanical properties.

The percentage and quality of the crystalline phase of a polymer strongly affect all of the material’s physical properties, including its strength and modulus, heat resistance, gas permeability, transparency or opacity and many others, thus strongly affecting the possible applications of a specific polymer.

Accordingly, the investigation and thorough understanding of the polymeric crystalline morphology, as well as their structures and the mechanisms of their formation, are crucial in obtaining the optimal desired properties of polymeric materials, as designed for specific applications.

When viewing polymeric crystals via polarized optical microscopy (POM) with crossed polars, the amorphous phase, which is commonly isotropic, appears as very dark or black regions of total extinction, and only the crystalline regions are clearly and brightly visualized, as the crystalline regions are anisotropic [1,2,3,4,5,6,7,8,9,10,11].

Polymeric spherulites, when viewed via POM, typically exhibit a quasi-central nucleation region, from which dense lamellar structures radiate outwards in a quasi-radial symmetry, up to the outer borders of the spherulite structure, which interacts with neighboring spherulites exhibiting a similar internal structure [1,2,3,4,5,6,7,8,9,10,11]. Nevertheless, the spherulites of some polymers (e.g., high-density polyethylene), when melt-crystallized under certain conditions (e.g., air cooling at room temperature), exhibit a concentric banded spherulitic morphology when viewed via POM [14,15,16,17,18,19,20].

Another widely known and extensively researched polymer crystalline morphology is the transcrystalline morphology. This morphology is formed in particular when a significantly high density of heterogeneous nucleation occurs, at a surface that is in direct contact with the polymer melt. Consequently, lamellar crystals or spherulites are densely nucleated on the heterogeneous surface, the growth of which is restricted in the lateral direction and occurs mainly in the direction from and normal to the heterogeneous nucleating surface [20,21,22,23,24,25,26,27,28,29,30,31,32,33,34,35]. This morphology is commonly observed at the fiber–matrix interface in semicrystalline polymeric composites and in semicrystalline polymers that crystallize in contact with a boundary, as in the case of injection molding or melt casting.

At any given suitable crystallization temperature, nucleation and subsequent growth on the heterogeneous nucleating surface start earlier than in the bulk matrix melt, allowing the formation of a significantly thick transcrystalline layer, before interaction with the adjacent growing front of the matrix crystals [20,21,22,23,24,25,26,27,28,29,30,31,32,33,34,35]. This can be explained by the following: when lowering the temperature of a polymer melt, at constant pressure, the free energy changes such that the phase transition of crystallization is energetically favorable. Nevertheless, under these conditions, nucleation and initial growth proceed via a metastable state, by which nuclei of the new crystalline phase appear and redissolve due to the surface energy of the newly created interface, which favors their dissolution [2]. Only nuclei above a critical size will grow to eventually form crystals. The time required to form stably growing nuclei is termed the induction time. The induction time increases non-linearly with an increasing crystallization temperature. The presence of a heterogeneous nucleating surface within the polymer melt significantly decreases the induction time, while concomitantly increasing the nucleation density. Thus, crystals nucleated in this way and growing generally perpendicularly outward from this surface (which, by definition, is a transcrystalline layer) start growing much earlier than the crystals nucleated in the bulk polymer melt. This always allows the formation of a significantly thick transcrystalline layer, before finally interacting with the neighboring growing spherulites in the bulk [20,26,27,28,29,33,34,35].

It was previously demonstrated in polyethylene-based composites [20] that when the crystallization conditions favor the formation of banded spherulites in the bulk matrix (concentric banding), the transcrystalline layer formed at the fiber–matrix interface also exhibits a banded morphology (this time, it is parallel and not concentric) [20]. Thus, the banded morphology is most probably not limited to any specific type of nucleation, whether heterogenous or homogeneous, or to any specific lamellar orientation (e.g., radial or parallel); it is rather related to the lamellar crystals’ growth process or, possibly, to periodic discontinuity stages during lamellar growth.

Throughout the existing related literature, the source of concentric banding in spherulites is consistently attributed to the multiple perpetual helical twisting of the lamellar crystallites around their axial direction [14,15,16,17,18,19]. This model has been consistently used in order to explain the concentric banded patterns of polymer spherulites viewed via POM. According to this model, lamellar crystallites nucleated at the spherulite center and arranged in radial symmetry within the spherulite periodically and helically twist in tandem to form these mostly perfectly circular, concentric banded patterns of spherulites [14,15,16,17,18,19].

It is important to note, though, that the vast majority of polymeric banded spherulites viewed via POM exhibit concentric, alternating, very bright (white or colored) rings interspaced by very dark to black rings of total extinction. As is well known in POM with crossed polars, total extinction (i.e., a very dark to black field or region) can only be obtained in an isotropic material, i.e., an amorphous phase, or in crystallites (lamellae) that are radially oriented in the same directions as the crossed polars—which is usually observed as a Maltese cross in each spherulite [1,2,3,4,5,6,7,8,9,10,11,12,13,14,15,16,17,18,19,20,21,22,23,24,25,26,27,28,29,30,31,32,33,34,35]. The abovementioned interspacing black rings mostly exhibit circular all-around total extinction, merging into the four dark to black extinctions of the Maltese cross and circularly exiting the Maltese cross portions while still maintaining the same, mostly uninterrupted dark to black total extinction all around [14,15,16,17,18,19,20]. This consistently observed circular all-around total extinction band in banded spherulites may be contradictory to the lamellar twisting model, since, even if all lamellae of the spherulite twist perfectly in tandem (which is highly improbable, since crystals are mostly imperfect, exhibiting defects), each of the radially oriented lamellae are at a different angle as related to the polarizers’ directions. Thus, at least some regions of each of the black rings should exhibit brightness and even color.

Due to the nano-scale of the lamellar thickness, the resolution range offered by POM cannot enable the investigation of the morphology and configuration of individual lamellae. Thus, the investigation of individual polymeric crystalline lamellar structures requires the use of scanning electron microscopy (SEM). Nevertheless, the viewing and investigation of the polymeric crystalline morphology and, in particular, their individual lamellar structures (in terms of viewing a completely isolated, exposed lamella) is strongly impaired by the amorphous phase enveloping all lamellar crystallites and filling all interlamellar gaps of the spherulites and the outer surfaces thereof. The amorphous material appears in SEM scans as a dark gray or black featureless mass, as opposed to the crystallites, which, when exposed, commonly appear as bright to white lamellar structures. Thus, throughout the relevant existing literature, no single complete lamella showing multiple clear, periodic helical twists has ever been isolated and viewed via SEM. Rather, most reported SEM scans of polymeric spherulites exhibit concentric rings of partially exposed edge-on lamellae (i.e., their side edge pointing upwards), interspaced by concentric rings of slight valleys, which are commonly filled with featureless amorphous material and which are often interpreted as the face-on regions of a lamellar twist, although there is no clear, unambiguous view of such twists. They are thus often accompanied by a schematic hand-made or computerized drawing, depicting a lamella exhibiting multiple perfect, periodic helical twists, as a possible interpretation of the SEM or POM image of the banded spherulites [14,15,16,17,18,19].

A periodic helical twist actually implies the repeated 360° twisting of the crystallite around one of its crystallographic axes, from the nucleation point along the entire lamellar length. Measurements performed on different semicrystalline polymers, using synchrotron microfocus X-ray diffraction, have revealed lamellar twisting amounting to 0.85°/μm in high-density polyethylene (HDPE) [36]—which would accordingly require a lamellar length of approximately 423 μm for the completion of a single helical turn (which is much larger than the diameter of an entire common HDPE spherulite—30–150 μm). They also showed only a twist of a quarter turn within approximately 25 μm and then continued growth without further twisting in isotactic polypropylene [37] (this would accordingly require a lamellar length of 100 μm for the completion of a single helical turn). As the commonly observed band width in banded spherulites is about 1–2 μm [14,15,16,17,18,19,20], these reported results stand in contrast to and are highly inconsistent with the commonly suggested lamellar twisting model of banded spherulite formation. As an additional example of the above-discussed literature, many investigators have proposed the continuous lamellar twisting model for poly(ethylene adipate) (PEA), which commonly shows a distinct banded ring pattern when viewed via POM [38]. However, Woo et al. [39,40,41] have more recently discovered that the interiors of the ring-banded PEA spherulites are composed of a discontinuous structure of aggregated blocks [39,40,41]. Tashiro et al. [42] investigated the crystalline morphology of the same polymer (PEA) via a synchrotron X-ray microbeam technique and concluded as follows: “Lamellae must not be assumed to be continuously twisted long plates spreading out over the spherulite. Rather, these lamellae should be considered to have limited areas of ~7 μm in length along the radial direction and with disconnected end zones” [42]. They also note, “the continuous twisting is not necessarily realized and/or not essential for banding behavior because discontinuity between the neighboring blocks exists” [42]; moreover, they further state, “At present, however, we have not found concrete reasons why these disconnected zones are created in the growth process of a large spherulite” [42].

It was previously demonstrated for a polyether triblock copolymer (Pluronic F-127) [35] that the amorphous non-crystallizable material that is excluded during spherulite growth and accumulates at its growing front inhibits the further diffusion of crystallizable molecules towards the growing front, causing a halt in the spherulite’s growth. It is concomitantly shear-oriented at the growing front interface, which induces the heterogeneous nucleation of an all-around transcrystalline ring. This process is repeated in sequential quasi-concentric rows until a collision with similarly outward-growing transcrystalline rows growing from and around neighboring central spherulites occurs. Thus, each resulting spherulite exhibited a central smooth morphology, which suddenly changed to a banded morphology at a certain radius and length of the spherulite [35]. These results indicate that banded spherulites can form via all-around sequential concentric transcrystallization. In view of these results [35], the above-described periodic discontinuity regions [39,40,41,42] most probably consist of intermittent self-shear-oriented amorphous bands between transcrystalline bands that are nucleated.

The present research investigates the mechanism of banded spherulite formation through the combined POM and SEM observation of very thin, two-dimensional semicrystalline polymer samples, obtained via the crystallization of very thin melt layers, thus non-destructively obtaining very thin cross-sectional slices of the banded morphologies in the planes of their initial central nucleation centers. It is hereby demonstrated in three different, well-known, semicrystalline polymers that sequential transcrystallinity is most probably the predominant mechanism of banded spherulite formation, heterogeneously nucleated on intermittent self-shear-oriented amorphous layers, formed at the crystalline–melt interface during the spherulites’ growth.

A profound, in-depth understanding of polymer crystalline morphologies and the mechanisms of their formation will facilitate improved industrial processing methods and material properties for the development of novel polymeric products and applications.

## 2. Experimental Section

### 2.1. Materials

Polyethylene glycol (PEG) (MW = 10,000) (Sigma-Aldrich Israel Ltd., an affiliate of Merck KGaA, Darmstadt, Germany, Rehovot, Israel).; high-density polyethylene (HDPE) Sclair 2909 (DuPont, Wilmington, DE, USA); Pluronic F-127 (Sigma); Kevlar fibers 149 (DuPont).

### 2.2. Instrumentation

Polarized optical microscopy (POM) studies were performed on a Nikon H550S optical microscope (Nikon, Tokyo, Japan) equipped with a C-SP polarizer (Nikon, Tokyo, Japan) and D-DA analyzer (Nikon, Tokyo, Japan), and an INVENIO 5SCIII 5M pixel USB3 color digital camera (DeltaPix, Smorum, Denmark), connected to the Deltapix software (Deltapix Insight 2.5). Small amounts of each polymer were molten between two identical clean glass microscope slides on a controlled-temperature heating plate. Increasingly strong pressure was manually applied downwards on the upper slide until the polymer melt spread in a very thin layer and the excess melt exited from between the slides via the slides’ edges. The slides were then separated by sideways sliding; thus, each of the two slides received approximately half of the already very thin melt layer, resulting in a very thin, sub-micron melt layer on each slide. The two slides were again placed on the heating plate for about 20 s in order to relieve any internal stress stemming from the pressing process. The melt on each of the slides was then crystallized under the chosen conditions and placed in the microscope’s viewing field. Alternatively, the two glass slides were not separated in samples intended for the examination of the effect of entrapped air bubbles or embedded fibers on the crystalline morphology and the induction of transcrystalline layer formation. Only the focus and light intensity were adjusted during the microscopy studies.

Scanning electron microscopy (SEM) studies were performed on a SEM JEOL 6510LV instrument (Tokyo, Japan) equipped with a secondary electron (SE) detector, with a resolution of 3 nm at 30 KV. The acceleration voltage was 10 KV. The crystallized samples were viewed with Au sputter coating. Microscope glass slides with very thin crystallized polymer layers were prepared as described above for POM. Each slide was cut in half with a diamond glass cutter so as to fit inside the instrument. The Au coating of the polymer thin films was performed along with the glass slide support.

Differential scanning calorimetry (DSC) analyses were performed on a Mettler STARe SYSTEM DSC3 (Mettler-Toledo LLC, Greifensee, Switzerland) equipped with a DSC sensor FRS 5 (Mettler-Toledo LLC, Greifensee, Switzerland) and an intracooler isolation kit. Small amounts of each of the polymers were accurately weighed (in the range of 5–15 mg) in an aluminum crucible and then sealed with a preliminarily perforated aluminum lid and placed on the sample site. An identical sealed aluminum crucible was placed on the reference site. The measurements were performed at a heating rate of 10 °C/min and under a nitrogen gas flow.

X-ray diffraction (XRD) measurements of each of the polymers were performed on a Panalytical X’Pert Pro diffractometer with Cu Kά radiation (λ = 0.154 nm). Full pattern identification was performed with the X’Pert HighScore Plus software package, version 2.2e (2.2.5), by Panalytical B.V. (Melvern, Worcestershire, UK) Phase analysis identification was performed by XRD at 40 kV and 40 mA. The XRD patterns were recorded in the 2Θ range of 5–50° (step size 00.2°; time per step 2 s).

## 3. Results and Discussion

The present research focused on investigating the mechanism of banded spherulite formation in three of the most well-known and researched semicrystalline polymers, namely high-density polyethylene (HDPE), polyethylene glycol (PEG) (MW = 10,000), and Pluronic F-127. These polymers were chosen for two additional reasons: (1) these polymers are known to produce relatively large spherulite crystals, as compared to most other common polymers, and (2) these polymers exhibit relatively low melting temperatures, as compared to most other common semicrystalline polymers, thus minimizing any possible effect of oxidation and/or thermal decomposition in the molten state, which may in turn also affect the crystalline degree and morphology.

**Figure 1:**
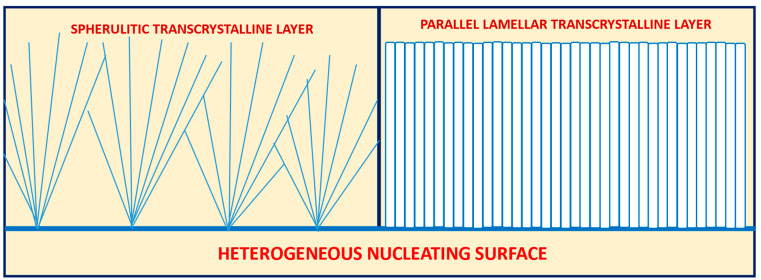
Schematic representation of the two types of transcrystalline layer morphologies: the spherulitic transcrystalline layer (**left**) and the parallel lamellar transcrystalline layer (**right**).

Based on numerous repeated morphological observations of semicrystalline polymers, the present research first defines two different types of possible transcrystalline morphologies. The first is a spherulitic transcrystalline layer, formed by the relatively dense nucleation of the spherulites’ nucleation centers on or adjacent to a heterogeneous nucleating surface. Consequently, their growth is significantly restricted in the lateral direction and they thus preferentially grow unidirectionally outward from the nucleating surface, exhibiting a layer with a parallel, partially closed, fan-like morphology on said nucleating surface. The second is the parallel lamellar transcrystalline layer, in which all lamellae constituting the transcrystalline layer are densely and adjacently nucleated on the nucleating surface, growing in parallel outward from the nucleating surface, forming a parallel, unidirectional, non-spherulitic layer morphology. Figure 1 exhibits a schematic representation of these two types of transcrystalline layers.

**Figure 2:**
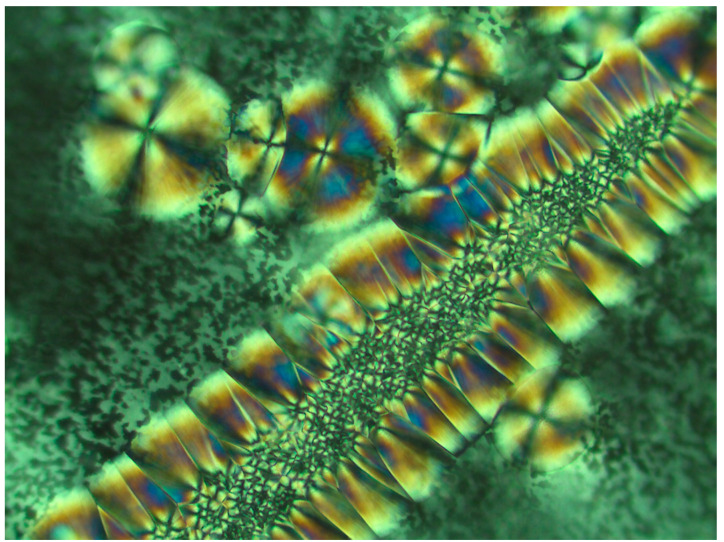
POM with crossed polars photograph exhibiting spherulitic transcrystalline layer in HDPE, nucleated and developed on shear-oriented polymer chains resulting from an occasionally occurring local melt flow prior to crystallization. This layer can be compared with the regular spherulites occurring in the adjacent polymer bulk. In the center region are densely nucleated transcrystals pointing upwards, the growth of which is restricted in view of the very thin, two-dimensionally prepared samples. Each component of the transcrystalline layer in the figure is nucleated on a black band of total extinction, indicating amorphous regions (this will be further discussed below) (magnification ×100).

**Figure 3:**
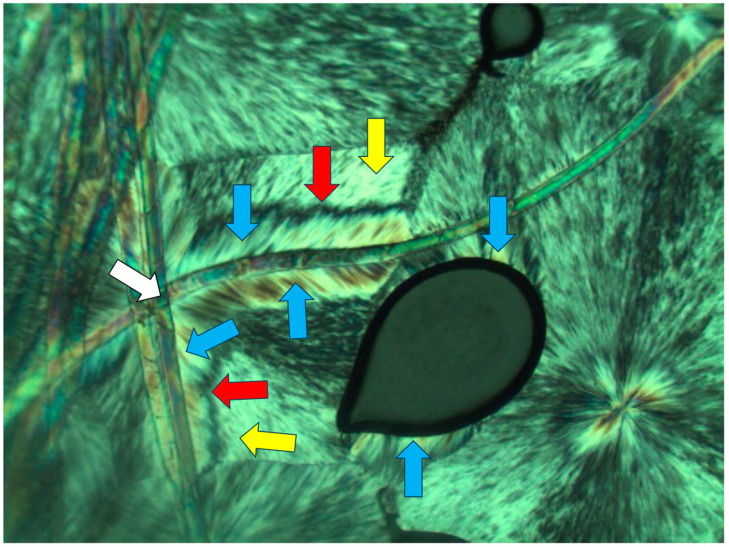
POM with crossed polars photograph exhibiting parallel lamellar transcrystalline layer (annotated with 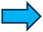) formed in Pluronic F127, nucleated on embedded Kevlar fibers (annotated with 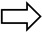). It is also important to observe the presence of a second transcrystalline layer (this time, it is dotted–spherulitic) above the first layer (annotated with 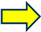), nucleated on a total extinction layer of amorphous material (black) (annotated with 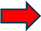); in addition, a parallel lamellar transcrystalline layer that developed on the surface of an occasionally occurring air bubble may be clearly observed (annotated with 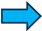) (magnification ×50). This will be further discussed below.

**Figure 4:**
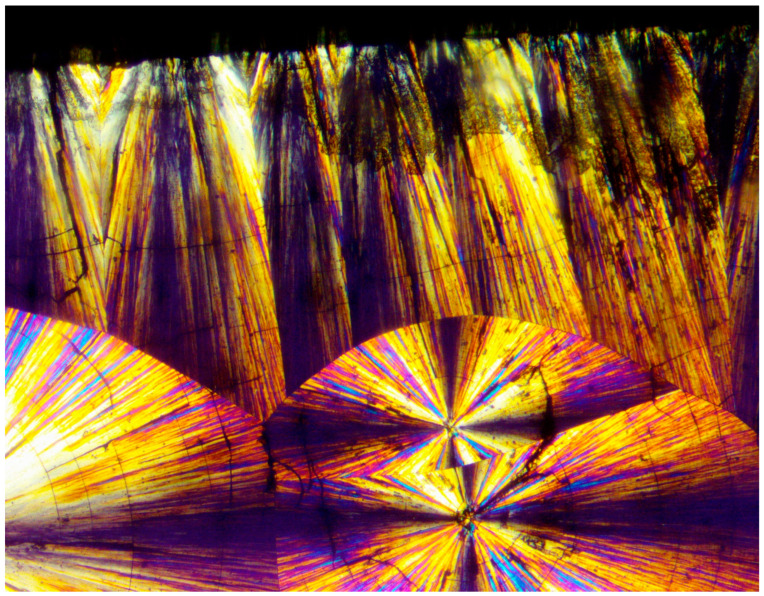
POM with crossed polars photograph exhibiting spherulitic transcrystalline layer in PEG10000, nucleated and developed on the outer edge of the material (black upper edge, which is the total extinction of air), most probably on the shear-oriented polymer chains due to the melt flow at the interface with air, occurring during the thin film sample’s preparation (most samples’ outer edges exhibited similar transcrystalline layer formation). This layer can be compared with the regular spherulites formed in the adjacent bulk polymer (magnification ×50).

Figure 2, Figure 3 and Figure 4 exhibit POM image examples of these two types, as defined above, in order to more clearly illustrate these morphologies, as they will be further discussed here below.

It can thus be deduced already at this stage that the heterogeneous nucleation of a transcrystalline layer does not necessarily require the presence of a solid crystalline nucleating surface; it can also be nucleated on amorphous self-shear-oriented polymer chains in the melt. It is observed that the self-shear orientation of the polymer chains in the melt can occur via the local melt flow in the bulk or a melt flow in contact with an interface—even in contact with the interface with air, e.g., in contact with an air bubble or at the edges of the sample.

Crystallizable polymers can form both types of transcrystalline layers, i.e., parallel lamellar and spherulitic. This was observed to mainly depend on the crystallization conditions, the type of heterogeneous nucleating surface, and the nucleation density [20,26,27,28,29].

Pluronic F-127 (also known as Poloxamer) is a hydroxy-terminated polyether triblock copolymer composed of a central hydrophobic chain of poly(propylene oxide) (approximately 65 repeating units) flanked by two hydrophilic chains of poly(ethylene oxide) (approximately 100 repeating units each), with a total molecular weight of approximately 12,500 g/mol. This well-known polymer has a vast range of applications in the biomedical, pharmaceutical, and cosmetic fields, as well as in the synthesis of multi-block copolymers. Although Pluronic F-127 is a semicrystalline polymer, only the PEG segments of the polymer can crystallize, while the PPO segment cannot crystallize due to the CH3 branches and thus this segment remains amorphous.

**Figure 5:**
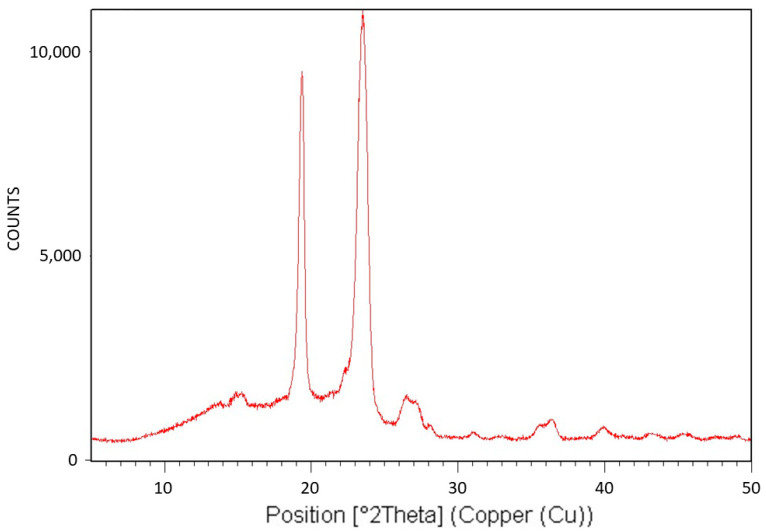
XRD pattern of Pluronic F-127.

Figure 5 exhibits the X-ray diffraction (XRD) analysis of Pluronic F-127. The XRD pattern is characteristic of the monoclinic crystalline structure of PEG [43] and is consistent with the fact that only the PEG segments of this triblock copolymer can crystallize. The relatively large diffuse scattering area beneath the peaks denotes very significant amorphous phase content, which is also consistent with the fact that the PPO segments of the polymer cannot crystallize. It is also important to conclude here that despite the significantly abundant presence of the non-crystallizable PPO segments of Pluronic F-127, although chemically bonded to the PEG segments, they only lower the degree of crystallinity of the PEG segments. They do not alter the characteristic PEG monoclinic crystalline structure and do not prevent their crystallization. Hence, it may be hereby deduced that these non-crystallizable segments, along with some non-crystallizable entangled PEG segments, are excluded during crystalline growth, constituting the polymer’s amorphous phase—the effect of which on transcrystalline layer formation will be further discussed here below. 

**Figure 6:**
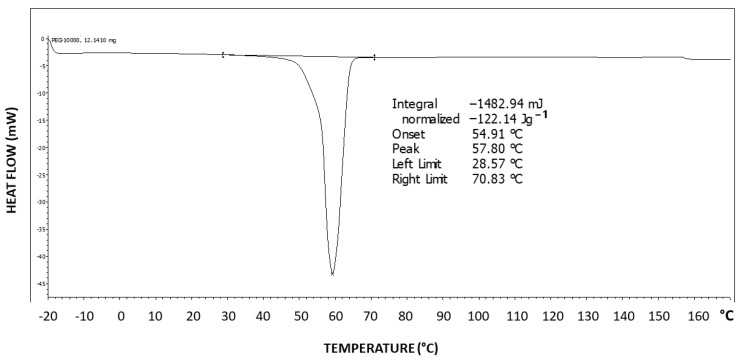
DSC thermogram of Pluronic F-127.

Figure 6 exhibits the differential scanning calorimetry (DSC) thermogram of Pluronic F-127. Two-step tailing to the left of the endotherm can be observed, indicating at least three types of crystal strength populations—two weaker types as tailings to the left and a stronger crystalline population represented by the peak at the right at 57.8 °C, which is highly characteristic of the PEG melting temperature [43]. The melting enthalpy hereby obtained is ΔH = 122.14 J/g.

The theoretical melting enthalpy for 100% crystalline PEG is ΔHm^0^ = 196.8 J/g [44]. Thus, the degree of crystallinity of Pluronic F-127 may be calculated by relating the melting endotherm enthalpy hereby obtained (ΔH = 122.14 J/g) to the ΔHm^0^ of PEG, resulting in a degree of crystallinity of 62% of the triblock copolymer, i.e., 38% of the polymer is amorphous, which is consistent with the observed significant diffuse scattering in the XRD pattern. By normalizing it to the fraction of the PEG segments in the polymer, a PEG segment degree of crystallinity of 88% is obtained (i.e., 12% of the PEG segments in the polymer have not crystallized), which indicates that the amorphous phase is mostly composed of non-crystallizable PPO segments but also of some non-crystallized PEG segments. 

**Figure 7:**
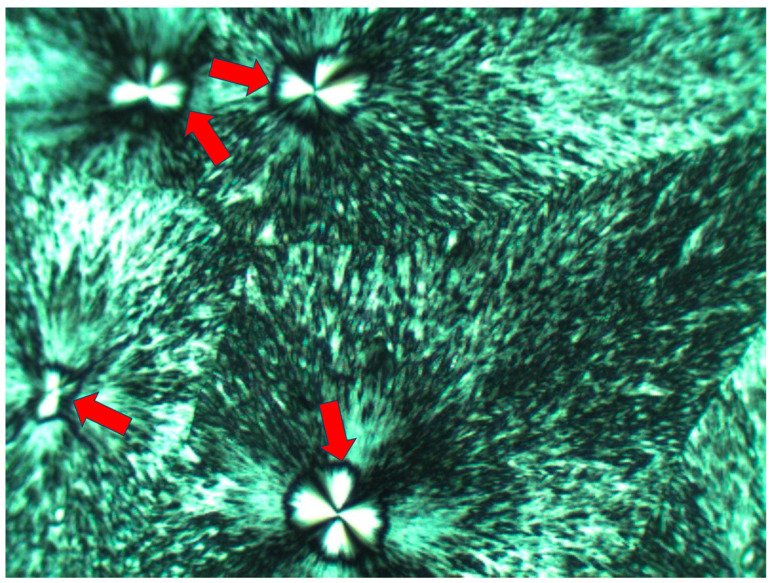
POM with crossed polars photograph of Pluronic F-127 crystallized at room temperature, exhibiting a very wide, dotted, concentric spherulitic transcrystalline layer morphology nucleated on an all-around amorphous layer (black rings—annotated with 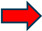) surrounding each smooth central spherulite.

Figure 7 exhibits the POM image of a thin layer of Pluronic F-127 crystallized from a melt at room temperature. Four smooth spherulites exhibiting Maltese crosses are observed, each surrounded by all-around black total extinction (annotated in Figure 7 with 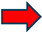), which in turn is surrounded by a very wide area of sequential dotted spherulitic transcrystalline layers developing outward until colliding with adjacent similar layers growing outward from the all-around total extinction surrounding the neighboring smooth spherulites (Figure 7). The fact that circular all-around total extinction is hereby observed between crossed polars unambiguously indicates that this region consists of an amorphous layer. Thus, the surrounding spherulitic transcrystalline layer around each spherulite has nucleated on this amorphous layer. This is also in strong agreement with the mechanism observed above in Figure 3, in which a second spherulitic transcrystalline layer formed by nucleation on an amorphous layer (black) on top of the first parallel lamellar transcrystalline layer is nucleated on the Kevlar fibers’ surface (Figure 3). Nevertheless, the concentric dotted array pattern of the transcrystalline layer indicates the presence of not only one transcrystalline layer but of many concentric, consecutive transcrystalline layers around each central spherulite.

**Figure 8:**
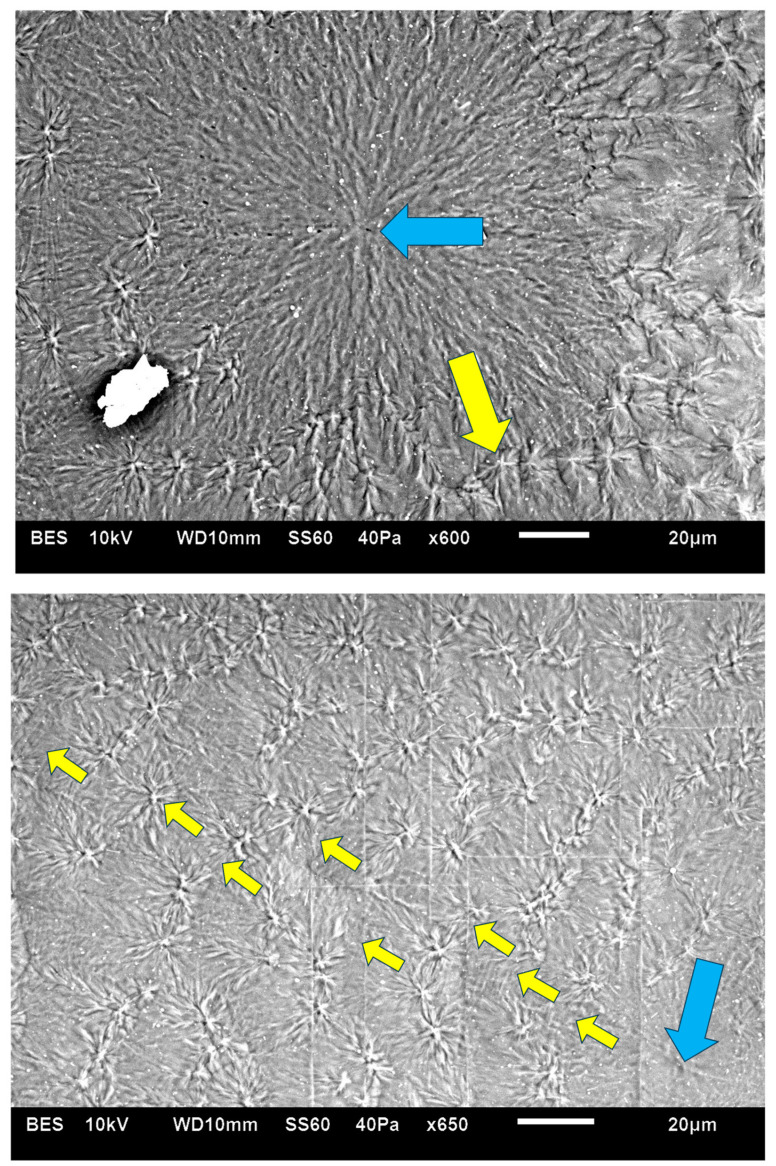
SEM micrographs of two representative regions of Pluronic F-127 crystallized at room temperature. The nucleation center of the central smooth spherulite is annotated with 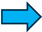. The inner border of the surrounding concentric spherulitic transcrystalline layers is annotated with 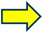.

Figure 8 exhibits an SEM micrograph of Pluronic F-127 crystallized at room temperature. The central smooth spherulite may be clearly observed, exhibiting the radial symmetry of lamellae nucleated in the center of the spherulite. Surrounding this central spherulite (the nucleation center of which is annotated with 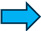) are sequential concentric rows of spherulitic transcrystalline layers of elongated small spherulites (annotated with 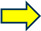), the first of which is nucleated around the central spherulite, and each following consecutive layer is nucleated on the previous one (Figure 8). It is important to note that, in Figure 8, the nucleation center of each small elongated spherulite in the concentric transcrystalline layers is nucleated at a distance from the edge of the central smooth spherulite or the edge of the previous transcrystalline row. This again indicates that the transcrystalline layers did not nucleate directly on the crystalline surfaces but nucleated on an amorphous layer covering these crystalline surfaces. The Pluronic F-127 samples exhibited significant sensitivity to charging during the SEM scans—this was most probably due to the high content of amorphous, non-crystallizable PPO segments, combined with the fact that the samples were very thin. Thus, SEM scans of Pluronic F-127 were performed at a low vacuum (40 Pa—which is annotated at the bottom of the SEM micrographs in Figure 8) in order to decrease the impact energy of the electron beam. Thus, some electron scattering occurred in the path to the detector. Consequently, the contrast here was lower, and the amorphous phase appears as a lighter gray rather than the dark gray to black in the two other polymers in this research. Nevertheless, all details are clearly seen. The edge-on lamellae exhibiting radial symmetry are very bright to white, the amorphous phase is light gray, and the borders between the spherulites within and between the transcrystalline layers are clearly seen.

The fact that, in POM with crossed polars, each of the small elongated spherulites in the concentric consecutive transcrystalline layers inherently exhibits a Maltese cross accounts for and explains the dotted patterned morphology viewed in Figure 3 and Figure 7.

**Figure 9:**
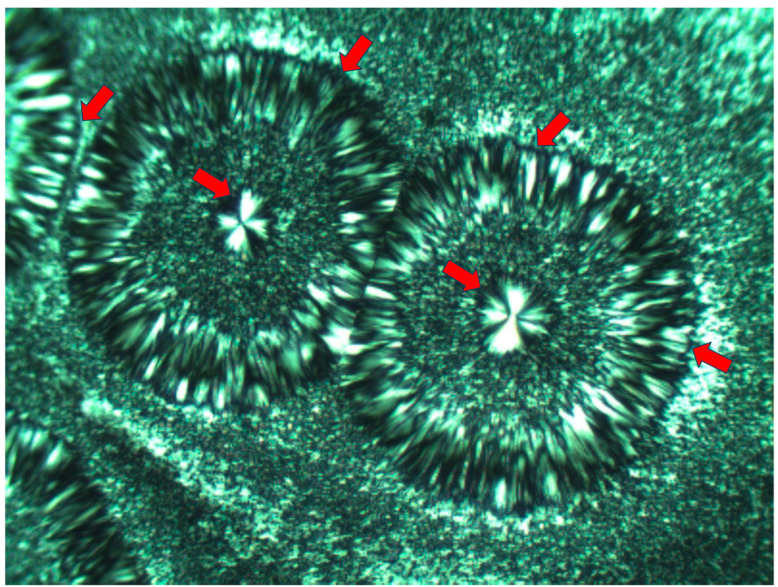
POM with crossed polars photograph of Pluronic F-127 crystallized at room temperature, exhibiting a very wide spherulitic transcrystalline layer morphology of significantly more elongated transcrystalline spherulites. Clearly visible regions with quasi-circular all-around total extinction of the excluded amorphous material on which the subsequent transcrystalline layer is nucleated are annotated with 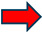 (magnification ×50).

Occasionally, one of the concentric transcrystalline bands may grow to a significantly larger width, consisting of significantly more elongated transcrystalline spherulites, as exhibited in Figure 9. Here, the quasi-circular all-around total extinction of the excluded amorphous material on which the subsequent transcrystalline layer is nucleated is clearly observed—some of these regions are annotated with 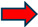 in Figure 9. 

These results are consistent with the mechanism previously demonstrated for Pluronic F-127 [35], indicating that the amorphous non-crystallizable material that is excluded during spherulite growth accumulates at its growing front, inhibiting the further diffusion of crystallizable molecules towards the growing front and causing a halt in the spherulite’s growth. The crystalline growth within the liquid viscous polymer melt inevitably exerts a shear force by which the chains in the adjacent melt are aligned, inducing heterogeneous transcrystalline nucleation—this process is repeated in sequential quasi-concentric rows, as more non-crystallizable material is excluded from each growing transcrystalline row and shear-oriented and a new transcrystalline row is concentrically nucleated and so on.

SAXS measurements of the Pluronic material reported in the literature [45] show that the remaining molecules in the disordered phase (i.e., in the Pluronic melt) are stretched during crystallization [45], which is highly consistent with the melt shear orientation mechanism described above and with the all-around extinction observed in the above figures, exhibiting the POM of Pluronic F-127.

PEG10000 is part of a large family of hydroxy-terminated polyethers, widely used in a vast range of applications, including in the pharmaceutical and cosmetic industries, as well as for the synthesis of higher block copolymers, especially for biomedical applications and artificial implant manufacturing.

**Figure 10:**
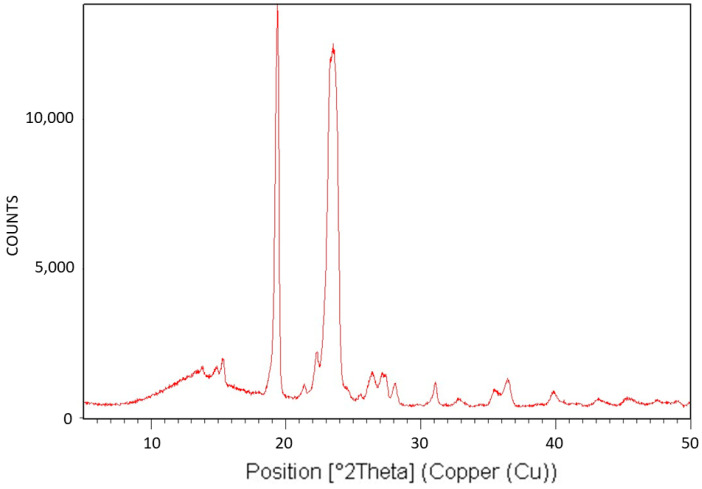
XRD pattern of PEG10000.

Figure 10 exhibits the X-ray diffraction (XRD) analysis of PEG10000. The XRD pattern is characteristic of the monoclinic crystalline structure of PEG [43]. 

**Figure 11:**
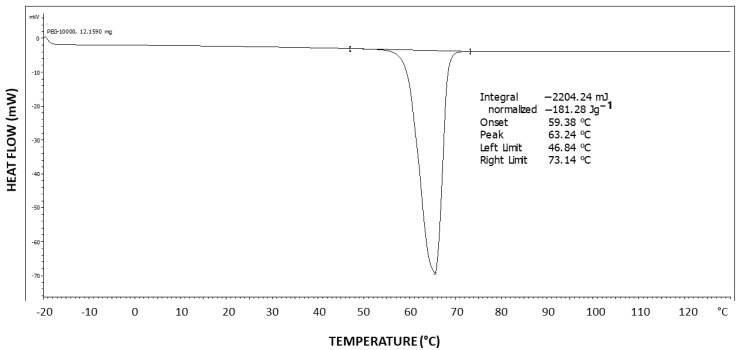
DSC thermogram of PEG10000.

Figure 11 exhibits the differential scanning calorimetry (DSC) thermogram of PEG10000. The theoretical melting enthalpy for 100% crystalline PEG is ΔHm^0^ = 196.8 J/g [44]. Thus, the degree of crystallinity of PEG10000 may be calculated by relating the melting endotherm enthalpy hereby obtained (ΔH = 181.28 J/g) to the ΔHm^0^ of PEG, resulting in a degree of crystallinity of 92% for PEG10000. Moreover, it is important to note the very symmetrical shape of the endotherm, with no tailing, which is in strong contrast with the DSC thermogram viewed above for Pluronic F-127, exhibiting two-step tailing to the left of the melting endotherm. 

PEG, and particularly PEG10000, mostly forms smooth, non-banded spherulites, as also seen in Figure 4 above. This is highly consistent with the very high degree of crystallinity obtained in the DSC analysis and the above-described mechanism, indicating that, when a less amorphous phase is present, there is less interference with the smooth, continuous growth of the spherulites. Nevertheless, as increasingly larger amounts of non-crystallizable material are excluded as a function of the crystallization time, spherulites that form at a later stage of the crystallization process tend to exhibit two and even multiple transcrystalline bands, nucleated on the shear-oriented amorphous material accumulated at the crystalline growth front surface. These bandings can be concentric or spiral, as will be discussed in the following.

**Figure 12:**
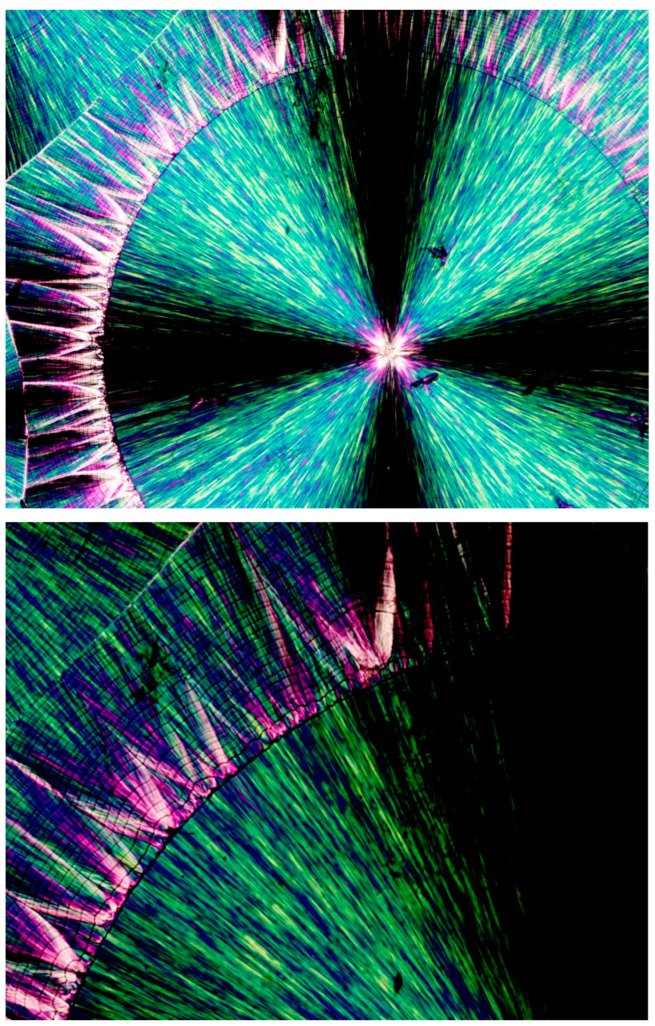
POM with crossed polars photograph of PEG10000, exhibiting a concentric banded spherulitic transcrystalline layer nucleated on the all-around black total extinction of the amorphous material (magnification ×100—**upper**; ×200—**lower**).

Figure 12 (upper photograph) exhibits the POM image of a PEG10000 spherulite, the growth of which was abruptly discontinued, followed by an all-around banded concentric spherulitic transcrystalline layer, apparently further completing the growth of the original central spherulite. At the much larger POM magnification of the same spherulite (Figure 12—lower photograph), it is clear that the concentric spherulitic transcrystalline layer did not nucleate on the central spherulite surface but on a significantly thick amorphous layer, exhibiting all-around black total extinction.

The fact that the transcrystalline layers’ nucleation and growth occur on polymeric fibers, as also seen in Figure 3, is widely known and is reported to occur due to the inherent axial extended-chain orientation in the polymeric fibers, which induces dense transcrystalline nucleation on their surfaces from the surrounding polymeric melt [20]. A very similar mechanism of transcrystalline nucleation was repeatedly and consistently observed in the present research, occurring on the shear-oriented polymer chains in the melt. This shear orientation-induced transcrystalline nucleation was mainly observed to occur at the interface boundary of the melt with a crystal growing front, at the interface boundary with air at the material edges, or with air bubbles entrapped within the melt.

It is well known in fluid mechanics and in plastics engineering and melt processing that a polymer melt that is flowing against an interface with a boundary will develop a flow velocity profile—with the melt flowing increasingly faster with the increasing distance from the boundary because of the friction of the melt with this boundary [46]. With the increasing distance from the boundary interface, the melt slides against itself more easily than against the boundary; thus, the flow velocity is gradually increased (generating a flow velocity gradient/profile). This phenomenon inevitably induces shear stress and even tensile stress in the melt flowing against the boundary, resulting in a polymeric extended-chain orientation adjacent to this boundary [46].

As was hereby consistently observed, this phenomenon also occurred at the interface boundary of the polymer melt with air, inducing transcrystalline nucleation and growth at the samples’ edges and at the interface with entrapped air bubbles in the melt (as seen above in Figure 3 and Figure 4). At the boundary with air, the shear stress orientation is caused by the melt flow against the surface tension of the polymer melt, created due to the abundant interchain bonding in the bulk melt, while there is no bonding at the interface contact with air (similar to the surface tension of water at the interface with air).

In a previous publication [29], it was shown that a transcrystalline layer could also form at the surface of an amorphous substrate (i.e., at the surface of a glass fiber in a HDPE matrix) when a shear force was applied by pulling the fiber in the HDPE melt, in order to induce molecular orientation in the melt adjacent to the fiber surface. A transcrystalline layer did not form in the un-sheared samples, which did not exhibit molecular orientation, but they consistently nucleated and formed on the shear-oriented molecules adjacent to the glass fiber surface [29]. It is also known that “glass fibers have no ability to induce crystallization at quiescent conditions” [47].

**Figure 13:**
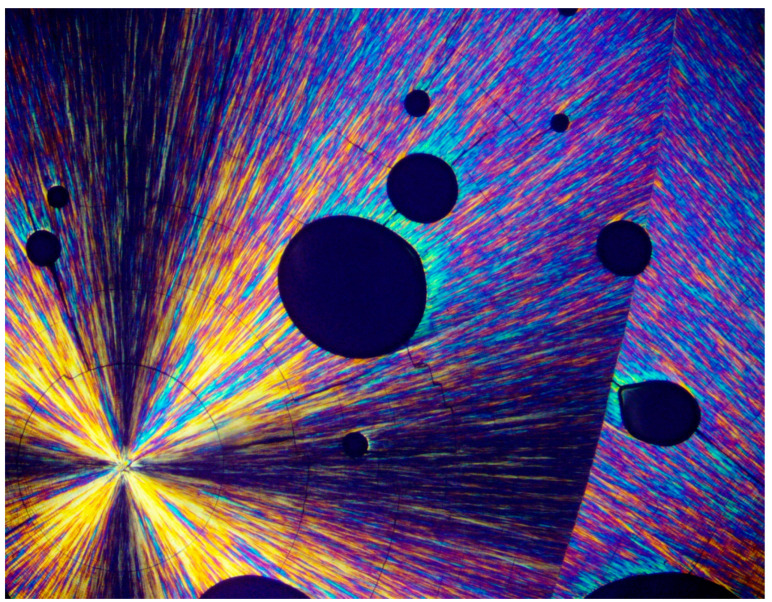
POM with crossed polars photograph of a representative example of PEG10000 spherulites, exhibiting transcrystalline lamellar nucleation and growth on entrapped air bubbles to form apparently complete spherulitic structures (magnification ×100).

Since an air bubble, by definition, contains nothing but air, the fact that a transcrystalline layer nucleates at the melt–air bubble interface is in complete agreement with the above-described shear orientation of the molten polymer chains at the interface boundary. Figure 13 clearly demonstrates this phenomenon, exhibiting a spherulite that encountered entrapped air bubbles during its growth process. Lamellar growth was halted when reaching an air bubble, but the spherulite growth continued in exactly the same lamellar direction via transcrystalline nucleation and growth from the opposite bubbles’ surfaces and beyond to form an apparently complete spherulitic structure. The black total extinction of the bubbles confirms that there is absolutely no other material inside the bubbles but air (Figure 13).

The hereby presented extended-chain melt shear orientation-induced transcrystalline nucleation mechanism is further confirmed by the observation that when the entrapped air bubbles are of an irregular shape, causing a turbulent melt flow (as opposed to a laminar flow), the transcrystalline nucleation and growth is highly disordered and clearly follows the multi-directional chain orientation induced by the turbulent melt flow.

**Figure 14:**
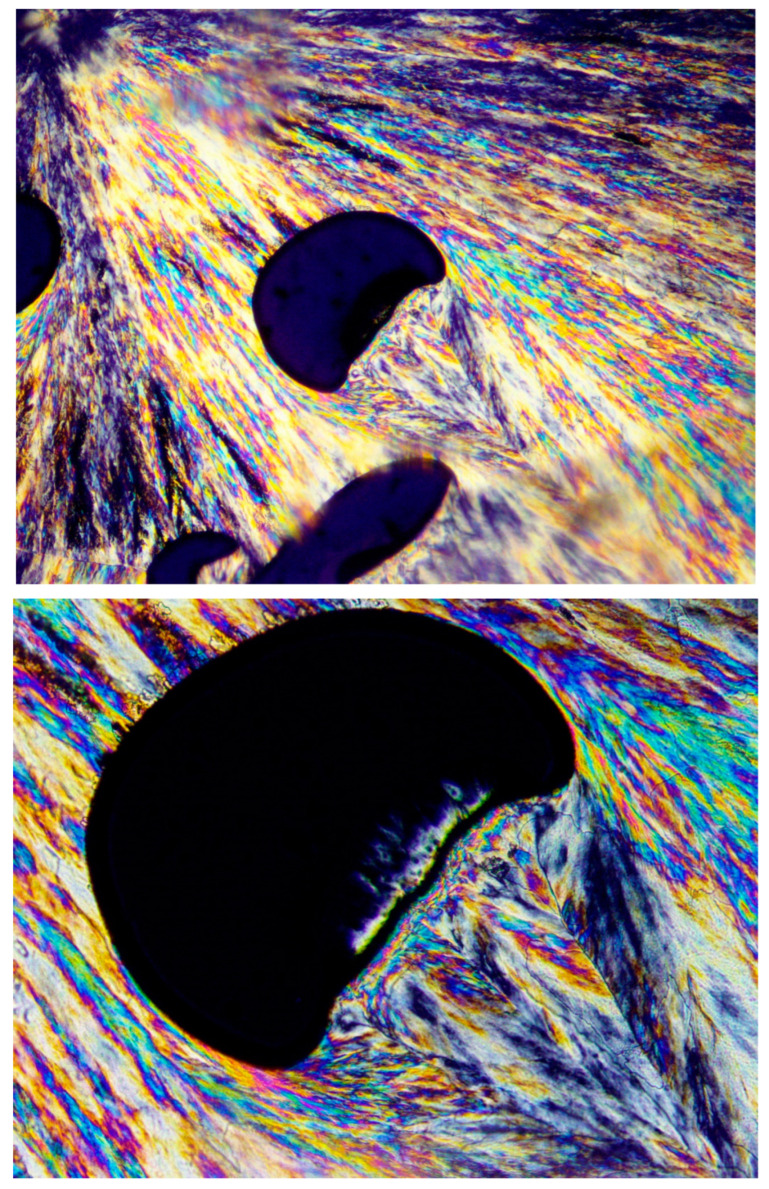
POM with crossed polars photograph of a PEG10000 spherulite, exhibiting a representative example of disordered multi-directional transcrystalline layer nucleation induced by a turbulent melt flow around an irregularly shaped entrapped air bubble (magnification ×100, **upper**; and ×200, **lower**).

Figure 14 exhibits a representative example of disordered multi-directional transcrystalline layer nucleation induced by a turbulent melt flow around an irregularly shaped entrapped air bubble. This strongly confirms the occurrence of transcrystalline nucleation induced by melt flow-derived chain orientation (Figure 14).

**Figure 15:**
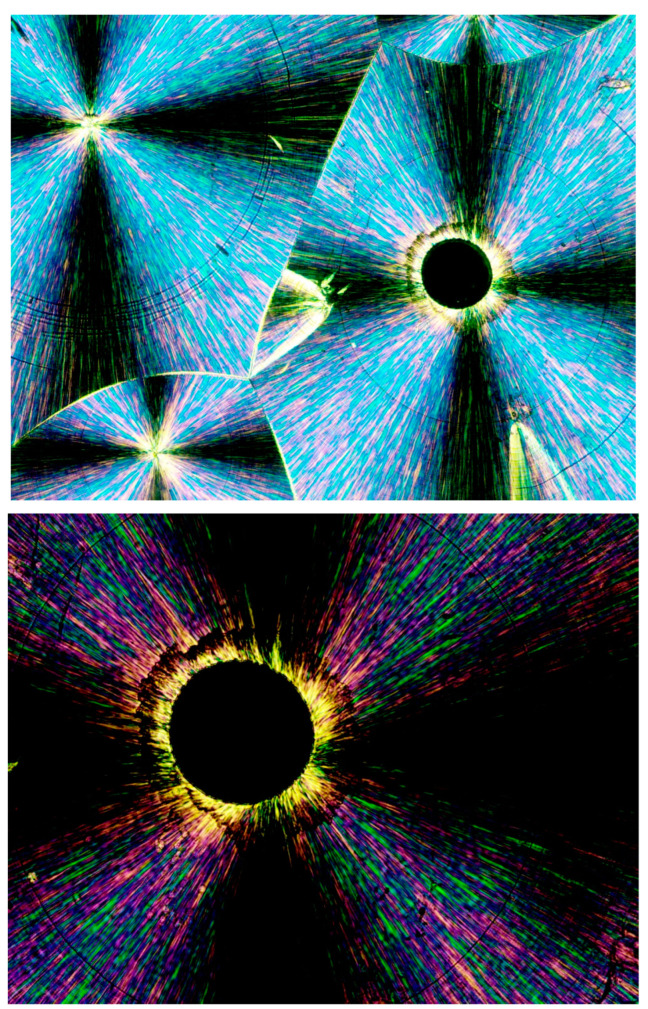
POM with crossed polars photograph of a PEG10000 spherulite, formed by all-around transcrystalline nucleation on the shear-oriented chains around a central air bubble (magnification ×50, **upper**; and ×200, **lower**).

It is also very interesting to note that an entire spherulite can form via all-around transcrystalline nucleation on the shear-oriented chains around a central air bubble, as presented in Figure 15. In this case, two-stage transcrystalline growth occurred, as seen in the higher-magnification area in the figure, also showing the presence of an all-around black total extinction amorphous layer between the two transcrystalline growth stages.

**Figure 16:**
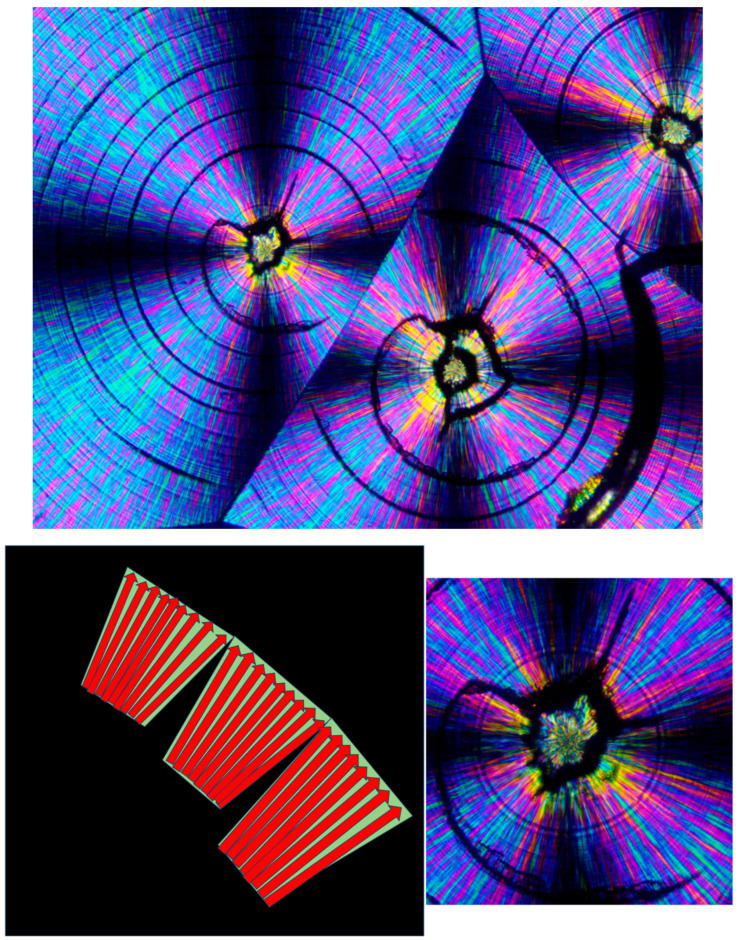
POM with crossed polars photograph of PEG10000 spirally bended spherulites (**upper**, magnification ×100); schematic representation of the transcrystalline mechanism of the black, sharp, ear-shaped amorphous region’s formation—the initial source of dislocation—finally resulting in the formation of spirally banded spherulites. (The transcrystalline layer’s representative lamellar growth direction is annotated with 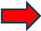; **lower**).

As also mentioned above, both concentrically bended spherulites and spirally bended spherulites can occasionally form in PEG. It is very interesting to note, though, that spirally banded spherulites were hereby consistently observed in PEG10000 to occur via transcrystalline nucleation on an asymmetrically shaped amorphous phase, enveloping one or two also asymmetrically shaped, initially formed crystalline clusters. Thus, the central regions of these spirally banded spherulites were hereby observed to consistently and almost identically exhibit an @-like shape with two cat ear-like shapes on top—exhibiting the black total extinction of the amorphous phase. The black, sharp, ear-shaped amorphous regions were formed by the sideways contact of orthogonally neighboring parallel lamellar transcrystalline layers, growing outward in a fan-like structure—hence their consistent occurrence. Figure 16 (upper) exhibits representative examples of these characteristic structures. Figure 16 (lower) also exhibits a schematic representation of the transcrystalline growth mechanism, enclosing the black sharp ear-shaped amorphous regions of total extinction, which are most probably the initial source of dislocation, finally resulting in the formation of spirally banded spherulites.

**Figure 17:**
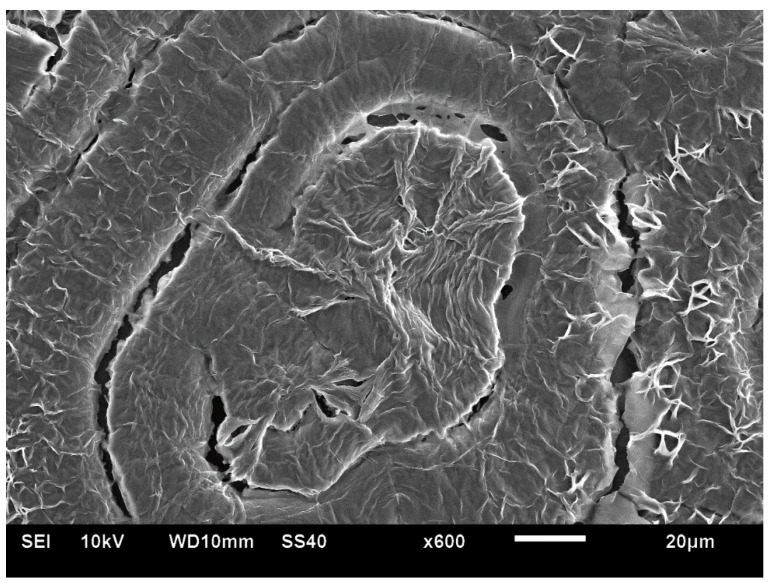
SEM micrograph of a PEG10000 spirally bended spherulite.

Figure 17 exhibits the SEM scan of a PEG10000 spirally bended spherulite. An initial, asymmetrical, small, central spherulitic sheaf structure can be clearly seen, exhibiting a nucleation center and sheaf structure with continuous edge-on lamellae (edge-on white) slightly protruding from the dark grey excluded amorphous material. The asymmetrical central structure encloses a mass of gray amorphous material, similar to that seen by POM. This morphology stops suddenly through a discontinuity. The following band exhibits a completely different lamellar structure consisting of highly branched lamellae; meanwhile, the following band exhibits yet another different morphology, mainly consisting of geometrical, polygonal-like, edge-on lamellar enclosures, enclosing excluded dark gray amorphous material. The morphologies of the consecutive bands are very different from the quasi-radial morphology of the asymmetrical central structure. These morphological discontinuities and the fact that all lamellae (whether highly branched or not) are edge-on—and absolutely no face-on lamellae are seen—may exclude the involvement of lamellar twisting. Moreover, as the various band widths vary greatly (both in POM and in SEM—Figure 15 and Figure 16), these bands cannot be the result of any regular periodic lamellar twisting.

High-density polyethylene (HDPE) is among the most applied and researched polymers worldwide, with a vast range of applications in almost every conceivable field, ranging from simple consumer products to biomedical applications.

**Figure 18:**
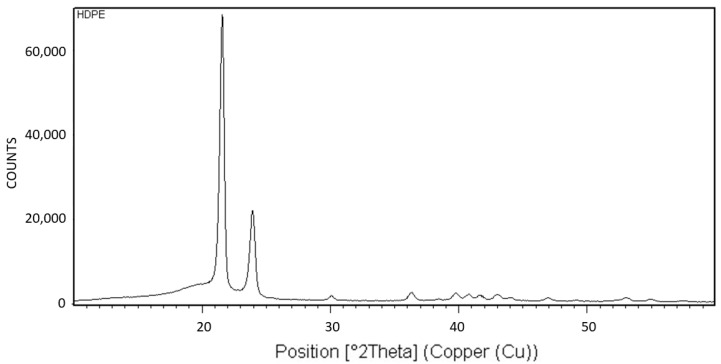
XRD pattern of HDPE.

Figure 18 exhibits the X-ray diffraction (XRD) analysis of HDPE. The XRD pattern is characteristic of the orthorhombic crystalline structure of HDPE [20].

**Figure 19:**
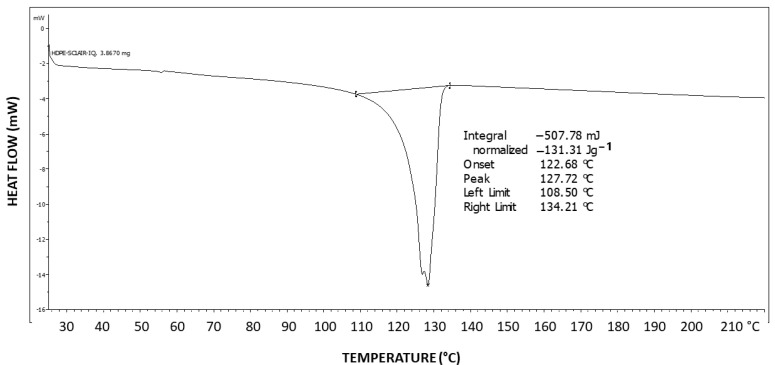
DSC thermogram of HDPE.

Figure 19 exhibits the differential scanning calorimetry (DSC) thermogram of HDPE. The theoretical melting enthalpy for 100% crystalline HDPE is ΔHm^0^ = 193 J/g [48]. Thus, the degree of crystallinity of the HDPE used here may be calculated by relating the melting endotherm enthalpy hereby obtained (ΔH = 131.31 J/g) to the ΔHm^0^ of PEG, resulting in a degree of crystallinity of 68% for HDPE. Thus, a significant amount of 32% of amorphous material is present in the polymer, which is excluded during the crystallization process and constitutes the amorphous phase of the polymer. 

**Figure 20:**
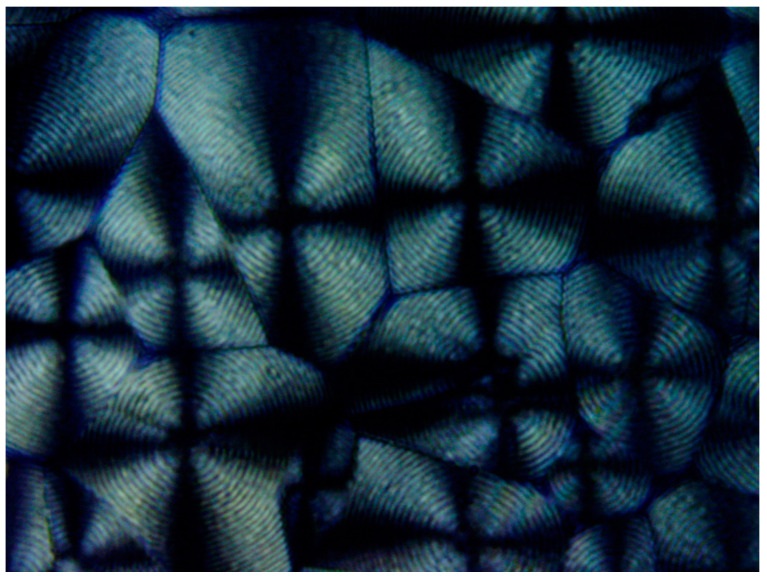
POM with crossed polars photograph of HDPE bended spherulites (magnification ×500).

Figure 20 exhibits a POM image of the HDPE banded spherulites. Multiple instances of very thin concentric banding are observed, with each spherulite also exhibiting a Maltese cross of black total extinction, indicating the crossed polars’ directions. The concentric bands consecutively alternate, with each two light to white concentric bands being separated by a black band of total extinction. The black total extinction bands completely merge and coincide with the total extinction in the four regions of the Maltese cross in each spherulite (Figure 20). Since polymer crystals (lamellae) can only exhibit total extinction when aligned in the directions of the crossed polars, the all-around black total extinction of the black bands in the spherulites most probably indicates that they consist of excluded amorphous material.

**Figure 21:**
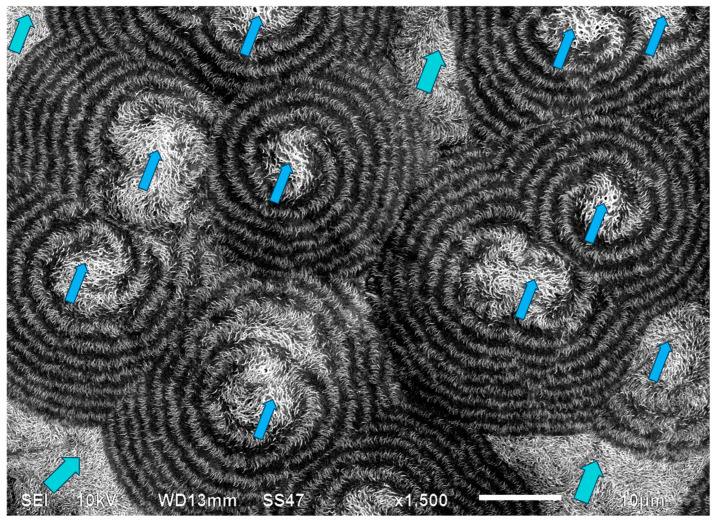
SEM micrograph of HDPE spirally bended spherulites. The non-spherulitic disordered lamellar structure in the center of each spherulite is annotated with 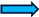. The same non-spherulitic disordered lamellar structure between some of the spherulites that are separated by a certain distance is annotated with 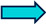. (Magnification ×1500).

Figure 21 exhibits an SEM scan of the HDPE bended spherulites. It can be clearly observed that these are in fact spirally banded spherulites. Moreover, it is important to observe that the center of each spherulite consists of a non-spherulitic disordered lamellar structure, very similar to the structures between some of the spherulites, which are separated by a certain distance. A bilateral black dislocation region within this disordered structure leads to the bilateral initiation of spiral banding. The spiraling bands all consist of parallel, unidirectionally oriented, edge-on lamellae, with the bands being separated and interspaced with spiraling black material. As also described in the Introduction, polymeric amorphous material appears in SEM scans as a dark gray or black featureless mass, as opposed to the polymeric crystallites, which, when exposed, commonly appear as bright to white lamellar structures. Thus, it may be concluded already at this stage that the featureless black areas in the SEM scan (Figure 21) are amorphous regions.

**Figure 22:**
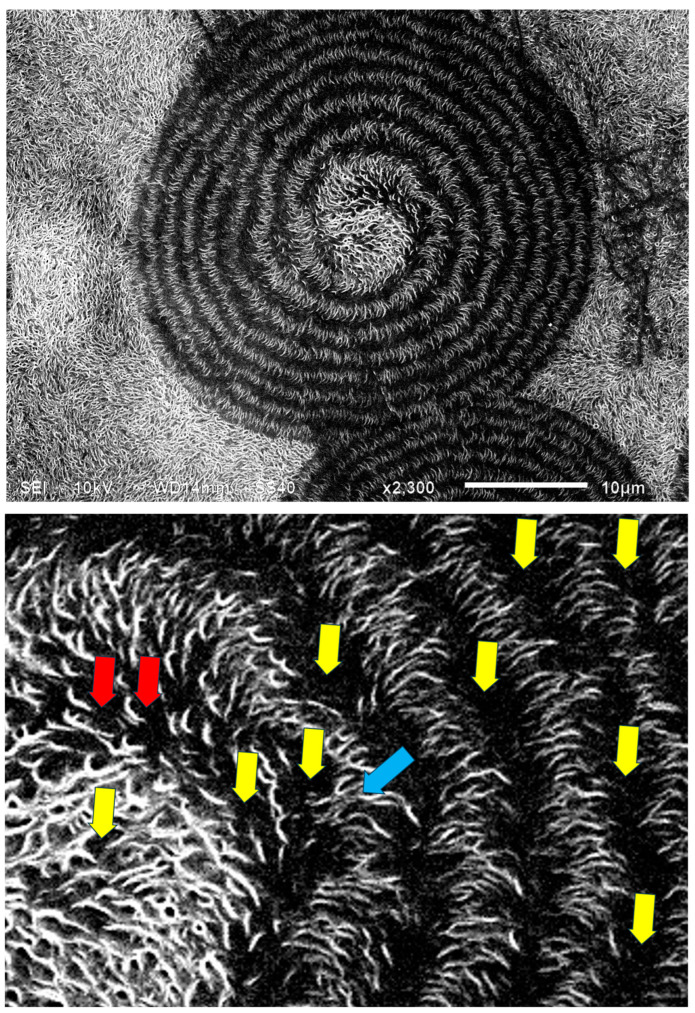
SEM micrograph of a HDPE spirally bended spherulite (**upper**) and an annotated detail of this SEM scan (**lower**). The excessive accumulation of amorphous material (black) and the consequent initial dislocation region is annotated with 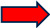. The point at which the purely transcrystalline parallel lamellar morphology starts is annotated with 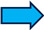, and some representative enclosed amorphous regions and the spiraling amorphous layer bands are annotated with 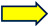. (Magnification ×2300).

Figure 22 exhibits an SEM scan of a single HDPE spirally bended spherulite at larger magnification (upper) and an annotated detail of this SEM scan (lower). As can be clearly observed in the annotated detail (Figure 22—lower), the non-spherulitic lamellar structure in the center of the spherulite consists of lamellae viewed edge-on, exhibiting three main shape types: (1) arched lamellae; (2) semicircular lamellae; and (3) circular lamellae (all three shape types may also be branched). All of these lamellar structures enclose black amorphous material in the interspaces between them; the circular lamellae also enclose black amorphous material inside (similar to bone marrow). At a certain growth stage, the excluded amorphous material accumulates in an excessively large amount, such that it is no longer enclosed between the lamellae and induces a bilateral dislocation in the growing lamellar structure (Figure 22—upper). This excessive accumulation of amorphous material (black) and the initial dislocation region are annotated with 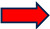 in Figure 22—lower. Since this amorphous material is not enclosed and is now in direct contact with the surrounding melt and also in contact with the crystal growing front, the amorphous polymer chains are most probably shear-oriented (as discussed above), which induces the nucleation of a parallel lamellar transcrystalline layer thereon. This transcrystalline layer consists of only parallel arched lamellae of approximately the same length (each neighboring parallel lamella being separated by black amorphous material). The point at which the purely transcrystalline parallel lamellar morphology starts is annotated with 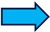 in Figure 22—lower. The transcrystalline layer progresses in a double spiral (due to the initial bilateral dislocation) as it is nucleated on a similarly spiral-shaped black amorphous layer. Some representative enclosed amorphous regions and spiraling amorphous layer bands are annotated with 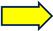 in Figure 21—lower. 

The distinct presence of the black amorphous material interspaced between the consecutive spiraling bands (Figure 20 and Figure 21) is highly consistent with the all-around black total extinction bands of the HDPE spherulites viewed here via POM (Figure 20).

**Figure 23:**
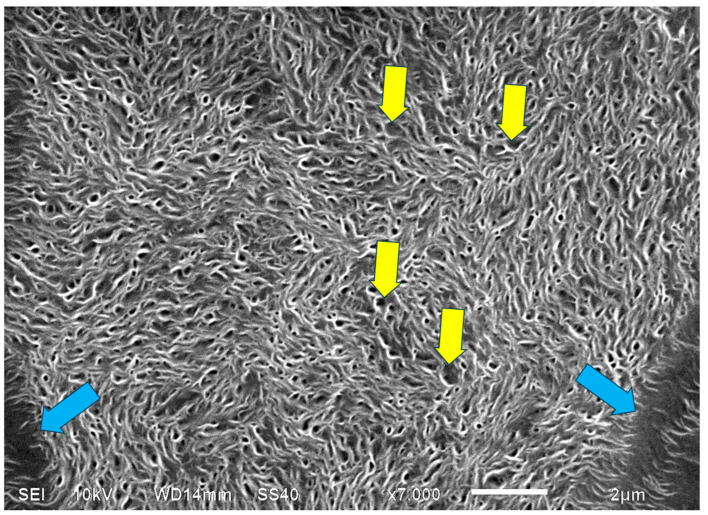
SEM micrograph of the non-spherulitic disordered lamellar structure between some of the HDPE spherulites that are separated by a distance. The edges of neighboring spherulites that are at a certain distance from each other are annotated with 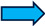; representative examples of enclosed interlamellar amorphous material are annotated with 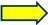. (Magnification ×7000).

Figure 23 exhibits a higher-magnification SEM scan of the non-spherulitic disordered lamellar structure between some of the HDPE spherulites that are separated by a certain distance. The morphology is very similar to the morphology observed in the center of each HDPE spherulite (Figure 21 and Figure 22), i.e., consisting of disordered edge-on lamellae, exhibiting the same three main shape types: (1) arched lamellae; (2) semicircular lamellae; and (3) circular lamellae (all three shape types may also be branched). All of these enclose black amorphous material in the interspaces between them. The circular lamellae also enclose black amorphous material inside (similar to bone marrow). It may be thus concluded that the regions that did not exhibit the excessive accumulation of amorphous material (which was not enclosed between the lamellae) did not induce the initial dislocation and subsequent spiral transcrystalline nucleation and thus did not result in the formation of the spherulite structures. It is suggested here that this non-spherulitic structure (at the spherulite centers and between distanced spherulites) is formed by vertical transcrystalline growth initiated on the chain orientation occurring on the surface of the glass slide substrate on which the molten polymer was crystallized (thus, these regions may appear disordered when viewed from above, but they are most probably significantly ordered in the vertical direction). The excessively excluded and shear-oriented amorphous material induced transcrystalline nucleation thereon, leading to the banded spherulites’ formation. In regions where the amorphous material remained isolated and enclosed within the lamellar structures, the transcrystalline nucleation in this amorphous phase was sterically hindered and did not lead to spherulite formation.

It is also important to note that the width of both the transcrystalline bands and the intermittent amorphous bands is approximately 1 μm, as seen in the SEM scans of Figure 20 and Figure 21. Measurements performed in the literature on HDPE, using synchrotron microfocus X-ray diffraction, revealed lamellar twisting amounting to only 0.85°/μm in high-density polyethylene [36], which would accordingly require a lamellar length of approximately 423 μm for the completion of only a single helical turn. Thus, the presently observed band widths of approximately 1 μm (Figure 21 and Figure 22) cannot be explained by or attributed to lamellar twisting. This is also supported by the observed amorphous phase bands between each pair of transcrystalline bands and the fact that no face-on lamellae are observed (Figure 21 and Figure 22), as well as the fact that these amorphous bands also exhibit all-around black total extinction in POM (Figure 20). This is also highly consistent with the discontinuous structure of the banded spherulites, as reported in the literature [39,40,41,42].

Since, in semicrystalline polymers, the amorphous phase is excluded during the growth of each crystalline lamella and accumulates at all lamellar surfaces and interlamellar spaces, semicrystalline polymers are considered as nanocomposites, with the amorphous phase being the matrix (the continuous phase) and the lamellar crystals being the reinforcement (the discontinuous phase) [12,13]. Thus, the morphology, size, and direction of the lamellar crystals within the amorphous phases of semicrystalline polymers may greatly affect their physical and mechanical properties. The present research reveals a newly observed transcrystalline morphology of banded spherulites and the mechanism of its formation, which may lead to new insights in the understanding and design of polymer processing for specific applications.

## 4. Conclusions

As discussed in the Introduction, the decades-long suggested paradigm of multiple periodic lamellar twisting being the source of polymeric banded spherulite formation was recently challenged by several different studies, the results of which strongly indicate that the lamellae are limited in length along the radial direction and have disconnected end zones. However, at present, there are no concrete reasons for which these disconnected zones are created in the growth process of a large spherulite.

The present research focused on presenting and demonstrating, in three different well-known semicrystalline polymers, that sequential transcrystallinity is most probably the predominant mechanism of banded spherulite formation, heterogeneously nucleated on intermittent self-shear-oriented amorphous layers located at the crystalline–melt interface during the crystals’ growth. Thus, the reason for the previously reported “disconnected zones that are created in the growth process of a large spherulite” is hereby also provided and explained.

Two types of transcrystallinity are hereby defined and presented: the spherulitic transcrystalline layer and the parallel lamellar transcrystalline layer.

It was hereby demonstrated that a transcrystalline layer can be nucleated and formed not only on a crystalline heterogeneous nucleating surface but also on amorphous self-shear-oriented polymer chains in the melt. It was observed that the self-shear orientation of the polymer chains in the melt can occur via a local melt flow in the bulk or a melt flow in contact with an interface—even in contact with the interface with air, e.g., in contact with an entrapped air bubble or at the edges of the sample. Spherulite lamellar growth stopped when encountering an entrapped air bubble but continued in the exact same direction beyond the same bubble via transcrystallization on the opposite side of the bubble’s outer surface, thus resulting in an apparently complete spherulite, as if no obstruction was present. When irregular entrapped air bubbles were present in the melt, inducing a turbulent flow, the resulting transcrystalline layers nucleated thereon followed the multiple directions and orientations of this turbulent flow.

In Pluronic F127, the morphological transition between the initial central, smooth spherulitic growth to a sequential spherulitic transcrystalline banded morphology occurred via the intermittent presence of the all-around black extinction of amorphous material, as viewed via POM, on which the transcrystalline bands were nucleated. The same phenomenon was also observed in PEG10000. Spirally banded spherulites of the same polymer were demonstrated to consistently occur via initial non-banded spherulitic growth, followed by a discontinuity consisting of an excessive amount of excluded amorphous material, which was further asymmetrically enclosed by subsequent transcrystalline growth thereon. This created the initial dislocation regions leading to the spiraling transcrystalline banding of the spherulites.

In HDPE, multiple instances of very thin concentric banding were observed via POM, with alternating light to white concentric bands being separated by black all-around bands of total extinction, indicating that they consisted of excluded amorphous material. This was also confirmed by the SEM scans, exhibiting spiraling transcrystalline bands consisting of parallel, unidirectionally oriented, edge-on lamellae, with the bands being separated and interspaced with spiraling black amorphous material. Spherulite growth starts with an initial period of non-spherulitic lamellar growth, consisting of apparently disordered lamellae viewed edge-on, enclosing black amorphous material in the interspaces between them. At a certain growth stage, the excluded amorphous material bilaterally accumulates in an excessively large amount, such that it is no longer enclosed between the lamellae and induces a bilateral amorphous discontinuity and dislocation—inducing double spiraling banded transcrystalline growth on a continuous spiraling band of amorphous material, in turn excluded by the growing transcrystalline bands. The hereby observed band widths of approximately 1 μm cannot be explained by or attributed to lamellar twisting. This is also supported by the observed amorphous phase bands between each pair of transcrystalline bands and the fact that no face-on lamellae were observed via SEM, as well as the fact that these amorphous bands also exhibit all-around black total extinction in POM. This is also highly consistent with the discontinuous (disconnected) structure of the banded spherulites, as reported in the literature.

In view of the fact that semicrystalline polymers are considered nanocomposites—with the amorphous phase being the matrix (the continuous phase) and the lamellar crystals being the reinforcement (the discontinuous phase)—the morphology, size, and direction of the lamellar crystals within the amorphous phases of semicrystalline polymers may greatly affect their physical and mechanical properties. The present research reveals a newly observed transcrystalline morphology of banded spherulites and the mechanism of its formation, which may lead to new insights in the understanding and design of industrial polymer processing for specific applications.

## Data Availability

Data are contained within the article.

## References

[1] Bower D.I. (2008). Introduction to Polymer Physic.

[2] Sommer J.U., Reiter G. (2003). Polymer Crystallization—Observations, Concepts and Interpretations.

[3] Muhukumar M. (2007). Shifting Paradigms in Polymer Crystallization. Progress in Understanding of Polymer Crystallization.

[4] Liu Q., Sun X., Li H., Yan S. (2013). Orientation-induced crystallization of isotactic polypropylene. Polymer.

[5] Hu W. (2023). Personal perspective on strain-induced polymer crystallization. J. Phys. Chem. B.

[6] Piorkowska E., Rutledge C.G. (2013). Handbook of Polymer Crystallization.

[7] Michael C., Zhang B.-H.G., Xu J. (2017). A Review on Polymer Crystallization Theories. Crystals.

[8] Pereira R.A., Mano E.B., Dias M.L., Acordi E.B. (1997). Comparative study on the lamellar crystal structure of high and low density polyethylenes. Polym. Bull..

[9] Crist B., Schultz J.M. (2016). Polymer spherulites: A critical review. Prog. Polym. Sci..

[10] Bassett D.C. (2003). Polymer Spherulites: A Modern Assessment. J. Macromol. Sci. Part B Phys..

[11] Bassett D.C. (2002). Polymer Spherulites, Encyclopedia of Materials: Science and Technology.

[12] Horniak G.L., Tibbals H.F., Dutta J., Moore J.J. (2009). Introduction to Nanoscience & Nanotechnology.

[13] Ozin G.A., Arsenault A.C., Cademartiri L. (2009). Nanochemistry, A Chemical Approach to Nanomaterials.

[14] Xu J., Guo B.H., Zhou J.J., Li L., Wu J., Kowalczuk M. (2005). Observation of banded spherulites in pure poly (l-lactide) and its miscible blends with amorphous polymers. Polymer.

[15] Lugito G., Woo E.M., Zunita M., Wenten I.G. (2019). Probing the interior lamellar periodicity and nano-assembly of polymer spherulites via combinatory etching methodology. Polymer.

[16] Xu J., Ye H., Zhang S., Guo B. (2017). Organization of Twisting Lamellar Crystals in Birefringent Banded Polymer Spherulites: A Mini-Review. Crystals.

[17] Su C.C., Lin J.H. (2004). Ringed spherulites in ternary polymer blends of poly(-caprolactone), poly(styrene-co acrylonitrile), and polymethacrylate. Colloid Polym. Sci..

[18] Sasaki S., Sasaki Y., Takahara A., Kajiyama T. (2002). Microscopic Lamellar Organization in High-Density Polyethylene Banded Spherulites Stidied by Scanning Probe Microscopy. Polymer.

[19] Bassett D.C., Hodge A.M. (1978). On lamellar organization in banded spherulites of polyethylene. Polymer.

[20] Stern T., Wachtel E., Marom G. (1997). Origin, Morphology and Crystallography of Transcrystallinity in Polyethylene-Based Single Polymer Composites. Compos. Part A Appl. Sci. Manuf..

[21] Quan H., Li Z.-M., Yang M.-B., Huang R. (2005). On transcrystallinity in semi-crystalline polymer composites. Compos. Sci. Technol..

[22] Liang Y., Zheng G., Liu S., Dai K., Liu C., Chen J., Shen C. (2013). β-Crystal in the iPP melt encapsulated by transcrystallinity and spherulites: Effect of molecular weight. J. Mater. Sci..

[23] Billon N., Magnet C., Haudin J.M., Lefebvre D. (1994). Transcrystallinity effects in thin polymer films. Experimental and theoretical approach. Colloid Polym. Sci..

[24] Liang Y., Liu S., Dai K., Wang B., Shao C., Zhang Q., Wang S., Zheng G., Liu C., Chen J. (2012). Transcrystallization in nanofiber bundle/isotactic polypropylene composites: Effect of matrix molecular weight. Colloid Polym. Sci..

[25] Li C., Gao Y., Wang L., Li J., Guo S. (2021). Fabrication, structure, and properties of Poly-(Lactide) multilayers with ultrahigh content, ordered, and continuous transcrystallinity. Polymer.

[26] Stern T., Teishev A., Marom G., Varelidis P.C., Papaspyrides C.D. (1996). Processing of Composites of Chopped PE Fiber-Reinforced PE Matrix. Adv. Compos. Lett..

[27] Stern T., Teishev A., Marom G. (1997). Composites of Polyethylene Reinforced with Chopped Polyethylene Fibers: Effect of Transcrystalline Interphase. Compos. Sci. Technol..

[28] Stern T., Wachtel E., Marom G. (1997). Epitaxy and Lamellar Twisting in Transcrystalline Polyethylene. J. Polym. Sci. Part B Polym. Phys..

[29] Zafeiropoulos N.E., Papaspyrides C.D., Varelidis P.C., Stern T., Marom G. (1999). Characterization of Coatings of LDPE Residual Matrix Deposited on Glass Fibers by a Dissolution/Reprecipitation Recycling Process. Compos. Part A Appl. Sci. Manuf..

[30] Zhang S., Minus M.L., Zhu L., Wong C.P., Kumar S. (2008). Polymer transcrystallinity induced by carbon nanotubes. Polymer.

[31] Lenes M., Gregersen Ø.W. (2006). Effect of surface chemistry and topography of sulphite fibres on the transcrystallinity of polypropylene. Cellulose.

[32] Klein N., Marom G., Pegoretti A., Migliaresi C. (1995). Determining the role of interfacial transcrystallinity in composite materials by dynamic mechanical thermal analysis. Composites.

[33] Wang C., Liu C.R. (1999). Transcrystallization of polypropylene composites: Nucleating ability of fibres. Polymer.

[34] Karger-Kocsis J. (2000). Interphase with Lamellar Interlocking and Amorphous Adherent—A Model to Explain Effects of Transcrystallinity. Adv. Compos. Lett..

[35] Stern T. (2017). Polymeric Micro-Sequential Concentric Transcrystalline Morphology Self-Assembly, with Intermittent Self-Shear-Oriented Amorphous Layers. Polym. Adv. Technol..

[36] Ratner S., Moret P.M., Wachtel E., Marom G. (2005). New Insights into Lamellar Twisting in Transcrystalline Polyethylene. Macromol. Chem. Phys..

[37] Assouline E., Wachtel E., Grigull S., Lustiger A., Wagner H.D., Marom G. (2001). Lamellar twisting in α isotactic polypropylene transcrystallinity investigated by synchrotron microbeam X-ray diffraction. Polymer.

[38] Lotz B., Cheng S.Z.D. (2005). A critical assessment of unbalanced surface stresses as the mechanical origin of twisting and scrolling of polymer crystals. Polymers.

[39] Woo E.M., Wang L.Y., Nurkhamidah S. (2012). Crystal lamellae of opposite orientations by three-dimensional dissecting onto spherulites of poly(ethylene adipate) crystallized in bulk form. Macromolecules.

[40] Lugito G., Woo E.M., Hsieh Y.T. (2015). Transitional ring bands constructed by discrete positive and negative-birefringence lamellae packed in poly(1,6-hexamethylene adipate) spherulites. Macromolecules.

[41] Woo E.M., Lugito G. (2015). Origins of periodic bands in polysmer spherulites. Eur. Polym. J..

[42] Tashiro K., Yoshioka T., Yamamoto H., Wang H., Woo E.M., Funaki K., Murase H. (2019). Relationship between twisting phenomenon and structural discontinuity of stacked lamellae in the spherulite of poly(ethylene adipate) as studied by the synchrotron X-ray microbeam technique. Polym. J..

[43] Bailey F.E., Koleske J.V. (1976). Poly(ethylene oxide).

[44] Bistac S., Brogly M., Bindel D. (2022). Crystallinity of Amphiphilic PE-b-PEG Copolymers. Polymers.

[45] Zhang F., Stühn B. (2006). Composition fluctuation and domain spacing of low molar weight PEO–PPO–PEO triblock copolymers in the melt, during crystallization and in the solid state. Colloid Polym. Sci..

[46] Strong B. (1996). A Plastics—Materials and Processing.

[47] Su F., Ji Y., Meng L., Chang J., Chen L., Li L. (2018). Shear-induced precursors in polyethylene: An in-situ synchrotron radiation scanning X-ray microdiffraction study. Polymer.

[48] Li D., Zhou L., Wang X., He L., Yang X. (2019). Effect of Crystallinity of Polyethylene with Different Densities on Breakdown Strength and Conductance Property. Materials.

